# Maternal bodies and medicines: a commentary on risk and decision-making of pregnant and breastfeeding women and health professionals

**DOI:** 10.1186/1471-2458-11-S5-S5

**Published:** 2011-11-25

**Authors:** Karalyn McDonald, Lisa H Amir, Mary-Ann Davey

**Affiliations:** 1 Mother & Child Health Research, La Trobe University, 215 Franklin St., Melbourne, Australia; 2 Australian Research Centre in Sex, Health and Society, La Trobe University, 215 Franklin St., Melbourne, Australia

## Abstract

**Background:**

The perceived risk/benefit balance of prescribed and over-the-counter (OTC) medicine, as well as complementary therapies, will significantly impact on an individual’s decision-making to use medicine. For women who are pregnant or breastfeeding, this weighing of risks and benefits becomes immensely more complex because they are considering the effect on two bodies rather than one. Indeed the balance may lie in opposite directions for the mother and baby/fetus. The aim of this paper is to generate a discussion that focuses on the complexity around risk, responsibility and decision-making of medicine use by pregnant and breastfeeding women. We will also consider the competing discourses that pregnant and breastfeeding women encounter when making decisions about medicine.

**Discussion:**

Women rely not only on biomedical information and the expert knowledge of their health care professionals but on their own experiences and cultural understandings as well. When making decisions about medicines, pregnant and breastfeeding women are influenced by their families, partners and their cultural societal norms and expectations. Pregnant and breastfeeding women are influenced by a number of competing discourses. “Good” mothers should manage and avoid any risks, thereby protecting their babies from harm and put their children’s needs before their own – they should not allow toxins to enter the body. On the other hand, “responsible” women take and act on medical advice – they should take the medicine as directed by their health professional. This is the inherent conflict in medicine use for maternal bodies.

**Summary:**

The increased complexity involved when one body’s actions impact the body of another – as in the pregnant and lactating body – has received little acknowledgment. We consider possibilities for future research and methodologies. We argue that considering the complexity of issues for maternal bodies can improve our understanding of risk and public health education.

## Background

The aim of this paper is to generate a discussion that focuses on the complexity around risk, responsibility and decision-making of medicine use by pregnant and breastfeeding women. By medicines, we mean prescribed, over-the-counter (OTC) and complementary medicines [1].

The purpose of this commentary is to draw on both the biomedical and sociocultural perspectives in recognition that these are usually discussed separately. We hope our multidisciplinary approach can deepen the discussion of this issue. To set the scene, we consider risk in the context of medicine use and decision-making for pregnant and breastfeeding women and the role of the ‘good mother’ discourse. We then examine the evidence surrounding the use of medicines during pregnancy and breastfeeding from three perspectives: the first from biomedicine, the second from the health care professional and the third from women. We suggest that the perspectives of the biomedical and the expert health care provider are generally privileged by public health campaigns. The paper concludes with suggestions for future research and the most appropriate methodologies to explore this relatively uncharted area of health research.

### Risk for pregnant and breastfeeding women

Our modern society has become increasingly concerned with understanding, calculating, managing, reducing or eliminating the risks associated with everyday life [2,3] and it is within this context that pregnant and breastfeeding women have a social and moral responsibility to manage risk [4-6]. The perceived risk/benefit balance of prescribed and OTC medicine, as well as complementary therapies will significantly impact on an individual’s decision to use medicine. For the maternal body – women who are pregnant or breastfeeding – this weighing of risks and benefits becomes immensely more complex because they are considering the effect on two bodies rather than one. Indeed the balance may lie in opposite directions for the mother and baby/fetus.

Pregnancy and breastfeeding, while inherently very private events, attract vast public attention and scrutiny. Deborah Lupton wrote that “the pregnant woman is surrounded by a complex network of discourses and practices directed at the surveillance and regulation of her body” and that “risk is a central discourse” [7] (p. 60). Helman pointed out that all cultures share beliefs about the *vulnerability* of the mother and fetus during pregnancy and that this usually continues throughout the early postpartum or lactation period” [8] (p. 46, original emphasis). Medical technology has embraced this vulnerability and the use of technologies, such as ultrasound, has meant that the fetus has increasingly acquired an individual identity that is separate from the mother and that the intensification of the health and well-being of the fetus has sometimes resulted in the mother being viewed primarily as the “maternal environment” [7] (p. 62).

Yet, despite the separation of the mother and fetus, the mother is responsible for her fetus’ health and well-being. “Her body therefore, is constructed as *doubly* at risk and she is portrayed as *doubly* responsible, for two bodies” [7] (p. 63, our emphasis). In addition, Lupton points out that pregnant women are expected to be extremely attentive in monitoring their bodies to ensure the health of their babies is not threatened in any way [7]. This self-regulation is extended to include the expectation that pregnant women and one could argue “good mothers”, are vigilant in their attendance at antenatal appointments and undergo all medical tests and examinations suggested by their health care professionals.

None of this is surprising when one considers the thalidomide disaster of the late 1950s and early 1960s. Pregnant women were prescribed thalidomide for morning sickness until it was recognised that it was a potent teratogen resulting in deformities in thousands of babies [9]. Since this time, women have been given strong messages about the importance of maintaining their health and avoiding toxins that can transfer from mother to baby. Pregnant women are even cautioned against simple analgesics such as paracetamol. Deborah Lupton details how:

“women are told that as well as avoiding any consumption of alcohol and tobacco (and illicit drugs such as marijuana and cocaine), they have been advised to give up tea, coffee and cola drinks, avoid certain sugar substitutes, avoid spa baths, be wary of microwave ovens, not use electric blankets, avoid having diagnostic x-rays, be careful in using household cleaning products and insecticides and not take prescription or over-the-counter therapeutic drugs (even headache pills) if possible” [7] (p. 64).

The phenomenon of “intensive mothering” was identified by Sharon Hays [10] whereby women must mother their children intensively to ensure they are seen to be “good mothers”. More recent work has positioned this phenomenon as contemporary motherhood [11,12] and suggests it still holds considerable power in societies such as Australia, the US and the UK [13]. Intensive mothers are also risk averse in their parenting approach [5] whilst recognising that “professional support” is essential to risk management [4].

### The “good mother”

Most pregnant and breastfeeding women are significantly influenced by the discourse of the “good mother” (and, in turn, intensive mothering) which is widely discussed in the research literature [14-18]. In essence, good mothers protect their babies from harm and put their children’s needs before their own [19] – which includes pregnant women. On the other hand, “responsible” women take and act on medical advice – they should take the medicine as directed by their health professional. This is the inherent conflict in medicine use for maternal bodies. Women in our society feel that they are ultimately responsible for producing a “perfect baby” [20,21] and presumably feel responsible for maintaining optimum infant health by providing breast milk free of possible contaminants such as medicines. Others have taken this further, arguing the rights of the baby or fetus are always prioritised above the mother [22,23]. This has been linked to the shift in western cultures during the middle of the 20^th^ century where the optimum way to raise children requires a “good mother” who anticipates and adapts to their children’s needs [6,24-26].

Once pregnancy is confirmed, women are faced with a multitude of decisions and risk assessments. They must decide what to eat (and not eat), what to drink (and not drink), what tests they will undergo (and what actions will be taken if test results indicate abnormality), what type of birth they want, how they will feed their infant, and so it goes on. However, in making these decisions, women become solely responsible for the welfare of their fetus. As Lupton writes, “there is no such thing as 'no risk' in pregnancy, but it is ultimately the woman’s responsibility to ensure she has done all that she can to minimise risk” [7] (p. 76).

As a result of the risk discourse that is prolific around pregnancy, it is now regarded as a hazardous journey that requires a high level of expert surveillance as well as vigilance on behalf of the woman. However, one of the “implications of this discourse is that the woman who fails to heed expert advice is portrayed as posing a risk to her foetus” [7] (p. 66). The 20^th^ saw the rise of the “monster mother” discourse where women are accused of causing harm to their children and fetuses [27]. Tsing argues that this discourse has extended to women who choose to give birth without medical assistance, regardless of the reasons why women make such decisions [27]. Others have identified that this discourse reduces women to an “environment” [28-30] and that while society may consider it irresponsible for individuals to neglect their own health, it may be considered criminal for pregnant and breastfeeding women to place the health of their fetus or baby at risk [7]. Western societies' condemnation of women who are perceived to fail by not putting their unborn babies' or children's needs ahead of their own is indicative of women's social positioning and exemplifies the privileging of biomedical knowledge over the women’s own knowledge.

## Discussion

### Biomedical perspective

#### Need for medicines during pregnancy and lactation

Of course, the use of medicines in pregnant and breastfeeding women may sometimes be essential to maintain the health of the woman (and baby). Poorly managed epilepsy in pregnancy can result not only in harm to the mother, but an increased risk of miscarriage [9]. Untreated depression during pregnancy has been associated with increased caesarean sections and higher admissions to neonatal intensive care units [31], poor decision-making such as increased alcohol use and missed medical appointments [32], as well as difficulties in bonding with the baby [33]. Despite the low risk of birth defects associated with commonly used antidepressants [34], many women are reported to discontinue their treatment upon confirmation of pregnancy [35,36].

HIV infection is another example where treatments contribute to the health of the mother. Furthermore, treatment of HIV during pregnancy has, potentially, another beneficiary: the baby. Use of antiretroviral (ARV) medicine during pregnancy and as prophylaxis to the baby, in addition to other interventions, can significantly reduce mother-to-child transmission (MTCT) of HIV to less than two percent [37]. However, there are considerable side-effects associated with ARV medicine and there is a history of toxicity, morbidity and mortality associated with its use early in the HIV epidemic. This has led to considerable scepticism and concern resulting in reduced uptake [38]; pregnant women have been particularly cautious about ARV use [39,40].

It is not surprising that both clinicians and women find decision-making difficult, given the lack of safety data about medicine use in pregnant and breastfeeding women. In the past, women were often completely excluded from drug trials – the United States (US) Food and Drug Administration (FDA) started advocating for women to be included in 1993 [41]. Although women have been included in drug trials more recently, pregnant and breastfeeding women are usually still excluded. The reason given for this exclusion was to protect the child [9]. The result is that existing safety data are limited: whilst there are small case series of women who have taken a particular medicine in pregnancy or lactation, systematic long-term follow-up remains lacking.

Actual risks for medicine use during pregnancy and breastfeeding are small. The real number of medicines proven to be teratogenic – that is an agent that irreversibly alters growth, structure or function of the developing embryo or fetus [42] – remains fewer than 30 [43]. Looking at breastfeeding women, Anderson and colleagues reviewed all published case studies of adverse events in infants caused by medicines, prior to June 2002 [44]. They evaluated 94 papers describing adverse reactions in 100 infants, of which none were “definite”, 47% were “probable”, and 53% were “possible” [44]. A case report of neonatal death from maternal codeine use has since been reported [45]. Obviously caution is needed but, considering the numbers of breastfeeding women who take medicines, the risk is small.

It is important to note that drug transfer (from mother to child) is not the same in pregnancy and breastfeeding. Most drugs taken by women during pregnancy cross the placenta from the mother’s circulation to the fetus by simple diffusion – resulting in drug transfer to the fetal circulation up to 100% [46]. In contrast, the breastfed infant receives far less maternal medicine than the fetus does. Medicines in the mother’s circulation may transfer into milk, but usually only in small amounts: infant exposure to the drug is five- to ten-fold less than during pregnancy [47,48]. (For more information about drug transfer see additional file 1: Pharmacokinetics of medicines in pregnancy and breastfeeding).

### Health professionals’ perspective

#### Health professionals’ perception of risk

Health professionals must also assess risk for their pregnant and breastfeeding patients. Lyerly and colleagues found that risk perception affects medical decision-making in pregnancy, pointing out that the tendency for health care professionals has been to “pursue zero risk to the fetus, independent of the absolute size of the risk, of competing considerations, or of recognition that fetal risk exists in other acceptable contexts” [49] (p. 981). They cite the example of vaginal birth after caesarean (VBAC), where caesarean section may be promoted in order to reduce the risk of perinatal death, without considering that even for a primary vaginal birth or caesarean section there are risks to the infant. They also identified that the risks of intervening are given precedence over the risks of failing to intervene. For example, radiological investigations may be delayed in pregnant women because of perceived potential harm from x-rays, while the real risk of septicaemia from an undiagnosed ruptured appendix is ignored; fetal loss may be more than 30% after a perforated appendix [49]. The use of medicines is similar. Halting medicines in pregnancy, or avoiding them in lactation, can lead to worsening maternal and therefore child health. Lyerly and colleagues suggest that “It is the physician’s obligation not to *eliminate* risk, but to help patients weigh risk, benefit, and potential harm, informed by best scientific evidence and guided by a patient-centred ethic” [49] (p. 982).

Health professionals are expected to be knowledgeable about medicines, yet are unlikely to have received specific education about prescribing for pregnant and breastfeeding women. Many pharmacology textbooks do not support the use of medicines for breastfeeding women: “it is prudent only to expose the infant to such risks if it is absolutely essential” [50] (p. 412). A recent survey of general practitioners (GPs), conducted by one of the authors (LHA), found that some GPs advised women to avoid breastfeeding while taking medicines like metronidazole and ibuprofen, which are considered compatible with breastfeeding [51]. Furthermore, our research has shown that health professionals often rely on the safety ratings given to medicines in pregnancy when making decisions about prescribing for breastfeeding women [51]. If health provider knowledge is poor, it is not surprising that the general public has little understanding of medicine use for breastfeeding women.

#### Decision making by health professionals

Our research suggests that decision-making for health professionals appears to be a spectrum from a straight forward decision, such as treatment of mastitis, to a complicated one requiring multiple inputs and consideration [51]. We need to be aware that the focus of medicine use is generally about risks. We need to balance evidence of danger with reassuring evidence [49]. For example, while alerting health professionals about the need for caution with codeine in breastfeeding women [52], we can explain other options for analgesia and evidence for their compatibility with breastfeeding.

"Quality use of medicines" programs are active in many countries, yet have not addressed the use of medicines for women who are pregnant or breastfeeding. For example, in the UK, the National Collaborating Centre for Primary Care (NCCPC) has recently published a guideline on involving patients in decisions about prescribed medicines, yet fails to mention use of medicines in pregnancy or lactation [53]. Similarly, the Canadian online database of interventions to promote evidence-based prescribing and medicines use [54] makes no specific mention of issues relevant to women.

### Women’s perspectives

#### Women’s perception of risk

A few studies have examined pregnant and breastfeeding women’s risk perceptions of commonly used medicines. A Norwegian study found that most women overestimated the teratogenic risk associated with medicine use during pregnancy. Interestingly, they found that women with a high perception of risk were more likely to be older, more highly educated and primiparous, although over 80% of the women had used drugs during pregnancy (most commonly paracetamol, penicillin and medicine for reflux) [55]. Similarly, a study by Sanz et al. [56], concluded that overestimated risk perception among women and health professionals led to induced abortions of healthy and wanted babies. Women’s perceptions of risk of the use of antidepressants during pregnancy has also been explored with 87% of women mistakenly believing that antidepressant use during pregnancy increased the risk of congenital abnormalities [57].

Increasingly, it seems that our society expects a breastfeeding woman to be “pure”: her body and her milk should be free from any form of contamination [58]. It is not surprising then, that studies have found that prescribing medicine to breastfeeding women may lead to early cessation of breastfeeding or that a breastfeeding mother may be denied treatment due to the possible risk to her baby [47,59].

#### Women’s decision-making during pregnancy and breastfeeding

Recently researchers have examined the complexities surrounding women’s experiences of antenatal screening, for example pregnant women’s decision-making processes with regard to antenatal screening for Down syndrome [20]. The authors of this review of qualitative studies on the topic have dismissed the Theory of Planned Behaviour as a particular decision-making trajectory and suggested that the decision-making takes place in a complex framework [20]. They plan to test the framework using ethnography and choice modelling research [20]. Pregnant women considering antenatal testing are often confused by the estimates of risks they are given: the risk of having a baby with Down syndrome, the risk for miscarrying secondary to testing, and so on [20]. On the other hand, how much harder would it be to make decisions when the potential risk of taking medicine while pregnant or breastfeeding is not quantified? Furthermore, the risks of the alternatives are not stated; the potential hazards of infant formula are rarely considered [60,61]. Often, formula feeding is such a cultural norm that health professionals and families have trouble recognising that this is an artificial food, potentially contaminated with bacteria [61] and potentially leading to adverse child health outcomes [62].

Public health discourse has increasingly framed personal health choices as social and moral issues [6,63] and as one’s own responsibility to sustain one’s health [2,3,64]. We would extend this to pregnant and breastfeeding women and suggest that many women now feel responsible for producing and maintaining a healthy child. This increased responsibility has resulted in hyper-vigilant women going out of their way to avoid any possible toxins while pregnant or breastfeeding. With increased awareness of environmental contaminants on health [65], the list of potential impurities continues to increase.

### Areas for research

Decision-making around the use of medicines for pregnant and breastfeeding women is an under-researched area in both the biomedical/pharmacological as well as the social context. Although the importance of the health of mothers and babies should be self-evident, we believe this is a neglected area. Chris Mulford has argued that breastfeeding is *invisible* to the health care system and in her list of “blind spots” is medicine use for lactating women [66].

Pharmaceutical companies have traditionally avoided involving women of reproductive age in drug trials and, although women are now included in many trials, pregnant and breastfeeding women continue to be excluded. This appears to be an area that everyone wants to steer clear of. Yet, pregnant and breastfeeding women do have acute and chronic medical conditions that may require medicines. We can see at least three areas where research is needed: at the level of drug testing, at the level of health professionals who are responsible for prescribing and dispensing medicine and at the level of the general public – in particular the woman herself.

It is timely for pharmaceutical companies to consider ways of determining whether medicines are compatible with pregnancy and lactation. The US FDA has proposed major revisions to labelling of prescription drugs in order to provide better information about the effects of medicines used in pregnancy and breastfeeding [67]. The current letter category for risks of drug use in pregnancy is inaccurate and overly simplistic [67].

We need a better understanding of the issues for health professionals faced with decision-making when the medicine is prescribed for one body, but may impact on two. Where do they get their information? How helpful do they find the information? How can this be improved? Similarly, understanding the ways in which pregnant and lactating women want to receive information about risks of taking medicines is crucial. For example, would women appreciate the quantification of the risks of each possible adverse outcome? Would presenting numerical risk information as a “thousand person” graphic [68] be more useful than presenting as numbers or percent (such as 1 in 1,000; 0.1%)? Could the estimate of the chance of suffering *no* adverse outcome be more useful? Alternatively, would women prefer a description of the possible adverse outcomes (without quantification) or a comparison with everyday risks (such as the risk of a particular birth defect without any known exposure to a teratogen, or the risk of car accident)? Can we quantify the risks to the child for premature cessation of breastfeeding and the introduction of infant formula? Would women like to be provided with this information? In addition to what information and how women would like to receive it, who would they like to provide it? The general practitioner during the consultation? The pharmacist dispensing the medicine? Written information from a government department, health professional body or health institution? There is a dearth of knowledge about the type of information as well as the most appropriate and effective way to convey it to pregnant and lactating women but we would argue that information presented to women should be woman-centred.

Beyond prescribed medicine, little is known about how pregnant and breastfeeding women interpret risk and make decisions about the use of social substances such as nicotine and alcohol as well as illicit substances. Insight into the decision-making of pregnant and breastfeeding women can inform better public health messages, clinical practice and policy guidelines.

We suggest that qualitative methodologies are often appropriate to address many of these research questions. Best results will be achieved by conducting collaborative interdisciplinary research, combining medicine, pharmacy, public health, psychology, sociology and anthropology. We also need to include the woman herself, in conjunction with her family and consumer advocates [69]. Women may wish to play a more active role in decision-making (the “patient-empowerment model”, rather than the biomedical-educational model) [70]. Previous research has found that consumers value information that enables “an informed choice promoting their autonomy; [consumers reported that] it was reassuring and reduced concern, conflict and anxiety about whether the medicine was the right one for them; and it gave them confidence in taking medicines” [70] (p. 115) and this may also be true for pregnant and breastfeeding women.

## Summary

Health research in general focuses on the mother *or* the baby (usually it is the mother who gets lost). The *complexity* of living in a body where one's actions impact on another body has not been recognised and is under-researched. We are calling for the development of research that focuses on the maternal body. This is important because the themes of “purity” in pregnancy [71] and breastfeeding [58], seem to be gaining momentum and increasing people’s anxiety about what the maternal body is exposed to.

Women must deal with competing interests (hers and her baby's) when making decisions about medicine use in the pregnant and lactating body. However, when making such decisions, pregnant and breastfeeding women rely not only on the expert knowledge of their health care professionals but on their own experiences and socio-cultural understandings as well [7]. Women are likely to be influenced by their families, partners, their cultural and societal norms and expectations, but also by discourses of risk, responsibility and good motherhood.

We argue that considering these issues in the complexity of maternal bodies can *improve understanding* of risk perception and decision-making concerning medicine use for the population as a whole as well as providing a better understanding of the decisions made by pregnant and breastfeeding women. In addition to research on the safe use of medicines during pregnancy and lactation, *qualitative research* is needed to explore in-depth the quandaries that women and their health care providers face when medicine is indicated for pregnant and breastfeeding women. Understanding decision-making by women and by health professionals requires suitable study methods (as recommended by the review of antenatal screening for Down syndrome [20]).

While it may be helpful to conceptualise the “maternal body” when examining medicine use in pregnant and lactating women, it is *over-simplistic* to believe the issues are always the same. The answer is not one response for both pregnant and breastfeeding women. Responses must be considered separately for the pregnant woman and the breastfeeding woman. Research to help us understand the concerns of the women and health professionals will help with planning salient education programs for both groups.

## Competing interests

The authors declare they have no competing interests.

## Authors' contributions

The paper is the result of discussions between the three authors and COMPASS colleagues. KM and LHA drafted the paper. All authors contributed to and approved the final version.

## Supplementary Material

Additional file 1 **Pharmacokinetics of medicines in pregnancy and breastfeeding** A description of what happens to medicines in the body of women who are pregnant or breastfeeding. Click here for file
