# Supplementary material for: Maternal bodies and medicines: a commentary on risk and decision-making of pregnant and breastfeeding women and health professionals
Source: BMC Public Health. 2011 Nov 25;11(Suppl 5):S5. doi: 10.1186/1471-2458-11-S5-S5 (PMC3247028; doi:10.1186/1471-2458-11-S5-S5)
Supplement: Additional file 1 — Pharmacokinetics of medicines in pregnancy and breastfeeding A description of what happens to medicines in the body of women who are pregnant or breastfeeding. [file 1471-2458-11-S5-S5-S1.doc]

# Additional file 1

# Pharmacokinetics of medicines in pregnancy and breastfeeding

Although the placenta was thought to protect the fetus from harmful agents in maternal blood, “the thalidomide catastrophe in the 1960s made it evident that chemical compounds can cross the placenta and give rise to serious developmental toxicity” [1] (p. 364). Thalidomide exemplifies the important effect of timing of medicine use: upper limb anomalies (absence of long bones) was associated with drug use at 27-30 days post-conception, while days 30-33 were associated with lower limb anomalies [2]. In addition to birth defects, another concerning aspect of maternal medicine use in pregnancy is the later development of cancer in the offspring; diethylstilboestrol (DES) is the best-known example [1]. DES was prescribed to pregnant women between 1941 and 1971 in an attempt to prevent miscarriage [3]. Daughters of women prescribed DES were found to be at increased risk of developing a rare vaginal cancer [4].

The pharmacokinetics of drugs taken during lactation is quite different from those taken during pregnancy. There is no risk of birth defects and drug transfer into milk depends on a number of factors, for example, drugs that are highly bound to proteins in the mother’s plasma will be less likely to transfer into milk, because only a small proportion of the drug is free to cross the alveolar epithelium [5]. When choosing medicines for breastfeeding women, the medicine with the higher protein-binding is always preferable [6]. Some medicines have such a large molecular weight that they do not enter milk; heparin is one example [5]. It also needs to be determined if the drug is absorbed from the gastro-intestinal tract (GIT). For example, antibiotics like gentamicin need to be given intravenously or intramuscularly to the mother because of poor GIT absorption and thus will be poorly absorbed by the infant’s GIT; most of the dose will be passed in the infant’s bowel motions [5].

Passage of medicines from maternal plasma to breast milk is a dynamic process – drugs are entering and exiting in an active manner [7]. So the level in breast milk will usually drop as the maternal plasma level drops and it is rarely necessary to suggest that women express and discard milk [7]. Drugs with shorter half-lives are preferred, as mothers’ plasma levels will drop more rapidly, and therefore there will be less drug transfer into milk.

Another factor to consider is the physiology of the infant/child. Will the drug need to be metabolised by the immature liver or kidneys of a preterm two kilogram infant? Or is the child a well-developed 15 kilogram toddler? Furthermore, is the infant totally reliant on breast milk for its nutrition – or is the toddler having only one breastfeed each day? It cannot be assumed that drugs that are not safe in pregnancy are not compatible with breastfeeding [8].

# References

1. Myllynen P, Pasanen M, Pelkonen O: **Human placenta: a human organ for developmental toxicology research and biomonitoring** *Placenta* 2005, **26**:361-371.

2. Little BB, Gilstrap LCI: **Human teratology principles**. In: *Drugs and Pregnancy.* 2nd edn. Edited by Gilstrap LCI, Little BB. New York: Chapman & Hill; 1998: 7-24.

3. Fuller C, Saibil D: **Post-market surveillance of therapeutic drugs in Canada**. In: *Women and Health Protection.* Toronto, ON: Women and Health Protection; 2005.

4. Herbst AL: **Clear cell adenocarcinoma and the current status of DES-exposed females**. *Cancer* 1981, **48**(2 Suppl):484-488.

5. Hale TW, Kristensen JH, Ilett KF: **The transfer of medications into human milk**. In: *Textbook of Human Lactation.* Edited by Hale TW, Hartmann P. Amarillo, Texas: Hale Publishing, L. P; 2007: 465-477.

6. Riant P, Urien S, Albengres E, Duche JC, Tillement JP: **High plasma protein binding as a parameter in the selection of betablockers for lactating women**. *Biochem Pharmacol* 2006, **35**:4579-4581.

7. Hale TW: **Medications in breastfeeding mothers of preterm infants**. *Pediatr Ann* 2003, **32**:337-347.

8. Akus M, Bartick M: **Lactation safety recommendations and reliability compared in 10 medication resources.** *Ann Pharmacother* 2007, **41**:1352-1360.
